# Synergy between SDGs 12.3 and 2.1 in lower-middle-income countries through the lens of food waste and energy imbalance

**DOI:** 10.1038/s41598-025-01579-x

**Published:** 2025-05-23

**Authors:** Tomohiro Okadera, Kazuaki Tsuchiya, Tatsuya Hanaoka, Kazuya Nishina

**Affiliations:** 1 Regional Environment Conservation Division, National Institute for Environmental Studies, 16-2 Onogawa, Tsukuba, Ibaraki 305-8506 Japan; 2 Social Systems Division, National Institute for Environmental Studies, Tsukuba, Japan; 3 Earth System Division, National Institute for Environmental Studies, Tsukuba, Japan

**Keywords:** Food waste, Food energy balance, Sustainable development goal, National income, Lower-middle-income countries, Synergy, Environmental social sciences, Sustainability

## Abstract

Halving food wastage at retail and consumer levels by 2030 is a target for Sustainable Development Goal (SDG) 12.3. Although previous studies have indicated that the food wastage extent differs based on the national income level, the relevance of this relationship is debatable owing to the controversial quantification of food wastage, usually performed using two methods based on actual generation or gaps for human calorific requirements. Therefore, in this study, we aimed to investigate this issue by analyzing the correlation between food energy imbalance and per capita income using food wastage generation data for 51 comparable counties. The results revealed possible synergies between SDG 12.3 and the improvement of food security (SDG 2.1) in certain lower-middle-income countries. That is, the per capita food wastage in countries facing a food energy deficit (95 kg/year) is remarkably higher than that in countries that have resolved their food energy deficit (66 kg/year). We presume that prolonging the food shelf-life could be the key factor in linking SDGs 12.3 and 2.1. Furthermore, as the lack of reliable data in lower-middle-income countries hinders the verification of this synergy, we propose 19 lower-middle-income countries for future investigation to verify the synergy between SDGs 12.3 and 2.1.

## Introduction

Food wastage is a critical issue in a world with increasing food insecurity and environmental pressures^1^. Approximately 13% of the world’s food is lost after harvesting and before reaching retail markets, and 17% of the total food available to consumers is wasted at the household, food service, and retail levels; however, one in ten people worldwide suffer hunger^2^. Numerous studies have indicated that reducing food wastage is an effective approach for overcoming food insecurity^3–8^, and transparent quantification of food wastage is necessary for social implementation^9–12^. Halving per capita global food wastage at the retail and consumer levels and reducing food losses along the production and supply chains, including post-harvest losses, by 2030, is therefore a target of Sustainable Development Goal (SDG) 12.3^13^. Food loss and waste indices (https://unstats.un.org/sdgs/metadata/) are indicators being developed toward SDG 12.3. Food loss and waste definitions are complicated owing to their application for various purposes^7,11,13–16^; however, based on the definition for SDG 12.3^18^, we defined food loss in this study as the loss of edible food at the production, post-harvest, and processing stages and food waste as wasted food, including inedible parts, at the consumption stages^13–16^. Global datasets of food loss can be accessed via the food balance sheet (FBS) of FAOSTAT (https://www.fao.org/faostat/en/); however, reliable data on food waste are limited^7,17,15^.

Previous studies have presumed that food waste depends on the national development level; however, this is debatable^14,18^. Per capita food waste has been considered substantially higher in industrialized countries than in developing ones; therefore, tackling food waste is a challenge for developed countries^7,11,19,20^. However, other studies have found that the per capita amount of household food waste (HFW) is similar across high-, upper-middle-, and lower-middle-income countries and that the correlation between household waste and the per capita gross domestic product (GDP) and income is negligible^21,22^. Moreover, because HFW includes inedible parts^13,15,16^, a country whose staple food has a low portion of edible parts should be more likely to generate HFW. In addition, some studies indicated that high-quality diets, such as those involving more fruits and vegetables^8^, generate more HFW due to higher portions of inedible parts and higher perishability^23–25^. Additionally, consumer preference for perishable food possibly increases with rising incomes^26,27^. As the perishability of food affects its life span, foods with short shelf lives are more likely to be wasted^14,28^ because HFW depends on domestic food storage behaviors^29^. Various technologies such as refrigeration, freezing, drying, and canning have been developed to prolong food shelf life^30–32^, but because social implementation of such technologies is contingent on national affordability of the additional costs^33,34^, food shelf life may vary by country, even between countries having the same dietary habits.

Food waste has been typically quantified based on the mass or calorific (energy gap) value^6,35^. The mass-based approach (MBA) uses direct (volumetric assessment, waste composition analysis, mass balance analysis, counting/scanning, and food waste records) or indirect (modeling, food balance, use of proxy data, and use of literature data) measurements^7,13,14,21,35^, whereas the energy gap approach (EGA), categorized as an indirect measurement, calculates food waste as calorific surplus of food supply over the energy requirements for a human^36^. The MBA provides substantial data by manual collection of on-site information on food waste. However, this approach generally includes various biases (such as geographic and measurement biases) and limitations, and constant measurement is difficult^13,21,35^. In contrast, the EGA generates global comparable food waste data using statistical models. However, the findings are applicable only in countries that have an abundant food supply because a nation with an insufficient food energy intake yields a negative value in the analysis, which is either considered indicative of no food wastage^37^ or adjusted to a positive value^38^. Therefore, the EGA, when yielding a negative value, additionally considers the food energy imbalance (FEI), such as the prevalence of undernourishment, which is a target of SDG 2 (https://unstats.un.org/sdgs/metadata/files/Metadata-02-01-01.pdf).

Although food waste can be evaluated using mass or the calorific value, the relevance of these indicators for the generation of food waste remains unclear because the MBA and EGA have been developed separately and their results are difficult to compare owing to differences in and uncertainties of system boundaries^7^, such as those related to the definition of food waste or geographic and temporal biases (especially for the MBA). Consequently, fundamental questions remain, such as whether a nation with insufficient food energy intake generates no food waste and whether developed nations with abundant food supply generate comparatively more food waste. Recently, data on the mass of food waste, with bias correction and evaluation of confidence in estimates, for each country in the world were published^21^. In the present study, we attempted to identify the relationship between food waste and development level by analyzing the correlation between the mass of food waste and FEI for 51 comparable countries. We characterize the HFW of the target countries via grouping them by the national income level and FEI characteristics. We then focused on specific groups of countries that showed remarkable differences in the relationships between HFW and FEI to further analyze factors such as food edibility and shelf life and determine the differences in HFW generation. We also sought to identify factors that are likely to reduce HFW for these specific groups of countries and discuss possible synergies between SDG 12.3 and 2.1. Finally, we propose techniques for validating the synergy identified in this study for use in future research.

## Results

### Correlation between FEI and HFW

**Fig. 1:**
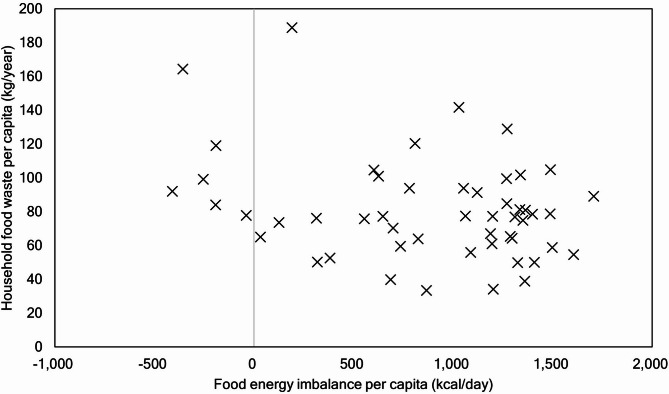
Correlation between food energy imbalance and household food waste.

FEI is proportional to the per capita GDP, as in Eq. (1). In practice, the global total per capita calorific demand tends to increase with the per capita GDP. Specifically, the countries with the highest per capita GDP should oversupply dietary calories owing to food waste^20^. Furthermore, the incentives to avoid food waste in these countries may be reduced in the consumption process because these countries can purchase food from global markets at an affordable price relative to the income^39^. This could account for the increase in FEI as the per capita GDP increases. To understand the primary relation between FEI and HFW, we analyzed the correlation between the per capita FEI estimated using Eq. (1) and the per capita HFW^21^ per country for the year 2019 (Fig. 1). A negative FEI indicates that the food energy supply does not reach the food requirements, whereas a positive FEI indicates the opposite. No significant correlation was observed between FEI and HFW at the national level; the coefficient of determination (R^2^) was 0.09. FEI was negatively correlated with food waste in a single regression with a p-value of 0.03 and a slope coefficient of -0.016 (Supplementary Data S1). High-income countries may thus generate less physical food waste than other countries but calorically waste more food.

### Data analysis based on HFW and FEI with income level

**Fig. 2:**
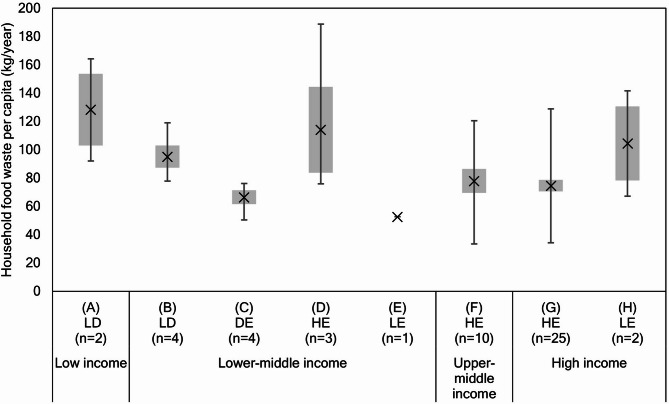
Average, standard error, maximum, and minimum values of food waste by country group. X denotes the mean value of household food waste generation, the gray-colored box shows the range of the standard error of the mean, and the whisker chart shows the range between the maximum and minimum values. On the horizontal axis, LD, DE, HE, and LE stand for “lower energy deficit of food,” “energy deficit to excess of food,” “higher energy excess of food,” and “lower energy excess of food,” respectively. “n” stands for the number of samples.

As the above analysis indicated a weak correlation, we additionally analyzed the relationships between FEI and HFW by grouping the target countries based on the income level and by considering FEI in the target year and temporal changes in FEI relative to the base year. The target countries were categorized into eight groups (Fig. 2): (A) low-income countries whose food energy deficit (FED) is improving but which are still facing an insufficient food energy supply compared with that in the base year, (B) lower-middle-income countries whose FED is improving, (C) lower-middle-income counties whose FED is solved but who had an FED in the base year, (D) lower-middle-income countries with increasing food energy excess (FEE) in the target year compared with that in the base year, (E) lower-middle-income countries whose FEE in the target year was lower than that in the base year, (F) upper-middle-income countries with increasing FEE in the target year, (G) high-income countries with increasing FEE in the target year, and (H) high-income countries whose FEE in the target year was lower than that in the base year. The average per capita HFW for group A (128 kg/year) was the highest, followed by that for groups D (114 kg/year) and H (104 kg/year). However, the representativeness of groups A and H is uncertain because they comprised only two samples.

The FEI for lower-middle income countries varied the most, with negative to positive FEIs (groups B–E), whereas the other countries had either negative (group A) or positive FEIs (groups F–H). In lower-middle-income countries (except group E that had only one sample), HFW decreased as FEI shifted from negative to positive because the average per capita HFW for group B (95 kg/year) is higher than that for group C (66 kg/year), whereas HFW increased again with increasing positive FEI values because the average per capita HFW for group C is lower than that for group D. Furthermore, Welch’s test^40,41^ (α = 0.05, two-sided; METHOD), indicated that HFW differed between groups B and C (*p* = 0.04), but not between groups C and D (*p* = 0.33), respectively .

In addition, among the groups in upper-middle-income and high-income countries (except group H owing to the above indicated uncertain representativeness), groups F and G did not show large differences.

### Data-based investigation on the primary influencing factor for the difference between HFW in groups B and C

**Fig. 3:**
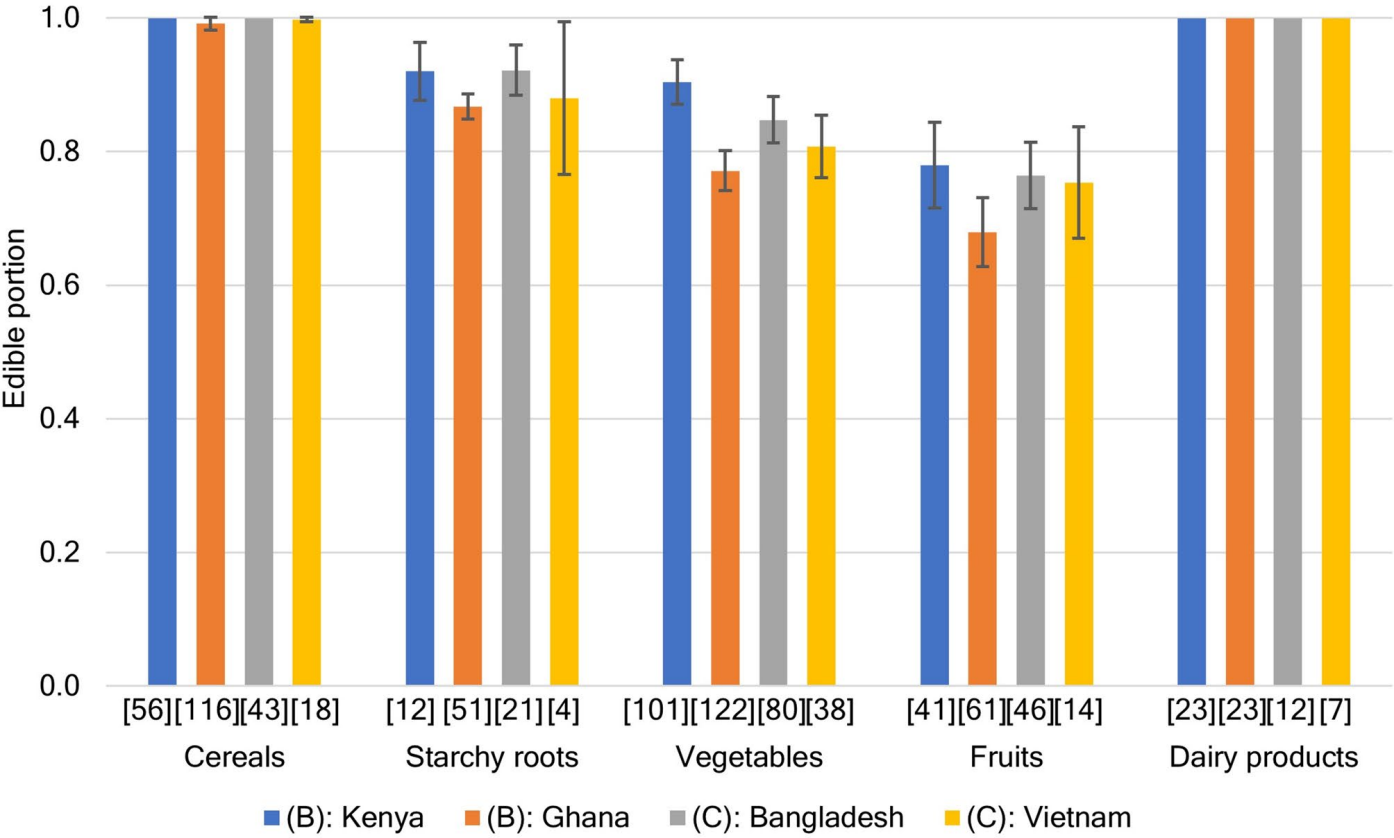
Average edible portion estimates of cereals, starchy roots, vegetables, fruits, and milk in countries for which data were available. Bar graphs show the mean value of item groups aggregated based on the edible portion of foods mentioned in the FCTs of the target countries, and the error bars show 95% confidence intervals. Sample sizes are shown as numbers in parentheses under the horizontal axis. The complete data for the figure are provided in Supplementary Data S2.

As shown in Fig. 2, the HFW of group B (Ghana, Kenya, Tanzania, and Zambia) was remarkably greater than that of group C (Bangladesh, India, Pakistan, and Vietnam); we attempted to identify the primary factor affecting this difference using the related statistical data. First, as food waste includes inedible parts, we presumed the difference in food edibility, namely the edible portion of the primary food, for group C to be higher than that for group B. The temporal difference in diets is negligible; food supply in South Asia and sub-Sahara Africa has changed only marginally over the past five decades^42^. A comparison between the FBS for groups B and C in 2019 revealed that cereals are the major common energy source for residents; however, group B is highly dependent on starchy roots and vegetable oils, whereas group C relies on diverse diets such as sugar and sweeteners, meat and animal fats, and vegetable oils, in addition to starchy roots. Simultaneously, based on the mass of supplied food (Supplementary Fig. S1 and Supplementary Table S1), where cereals (excluding beer) are the primary nutrient source, group B showed a relatively high dependency on starchy roots and fruits, whereas group C showed dependence on vegetables, milk (excluding butter), and fruits rather than starchy roots. As cereals, starchy roots, vegetables, fruits, milk, and their products are possibly the major constituents of food waste, their edible portion should be estimated to clarify the inedible portion for these countries. Food composition tables (FCTs) show edible portions of food available for different countries; among group B and C countries (https://www.fao.org/infoods/infoods/tables-and-databases/en/), edible portions of the total food were based on those in Kenya and Bangladesh^43,44^. We used the edible portion stated in the FCT for Western Africa for Ghana^45^ because Ghana does not have a national FCT. The latest FCT of Vietnam does not include an edible portion estimate (http://www.nutrition-smiling.eu/content/view/full/48718), but the Vietnamese FCT for 2007 includes the disposal ratio of food^46^, which was used to estimate the edible portion. Based on a dataset (Supplementary Data S2) of 898 available food items compiled from the four FCTs, we derived the mean edible portion estimate by food items (Fig. 3). In these countries, almost no cereals or dairy products have inedible parts, and fruits have the lowest edible portions (0.68–0.78 on average). In addition, the edible portion of starchy roots is higher than that of vegetables. Therefore, the edible portion of starchy roots, which group B highly depends on, is higher than that of fruits and vegetables, which constitute the primary food of group C. Therefore, the differences in HFW between groups B and C may not be caused by the edible or inedible portions of diets.

**Fig. 4:**
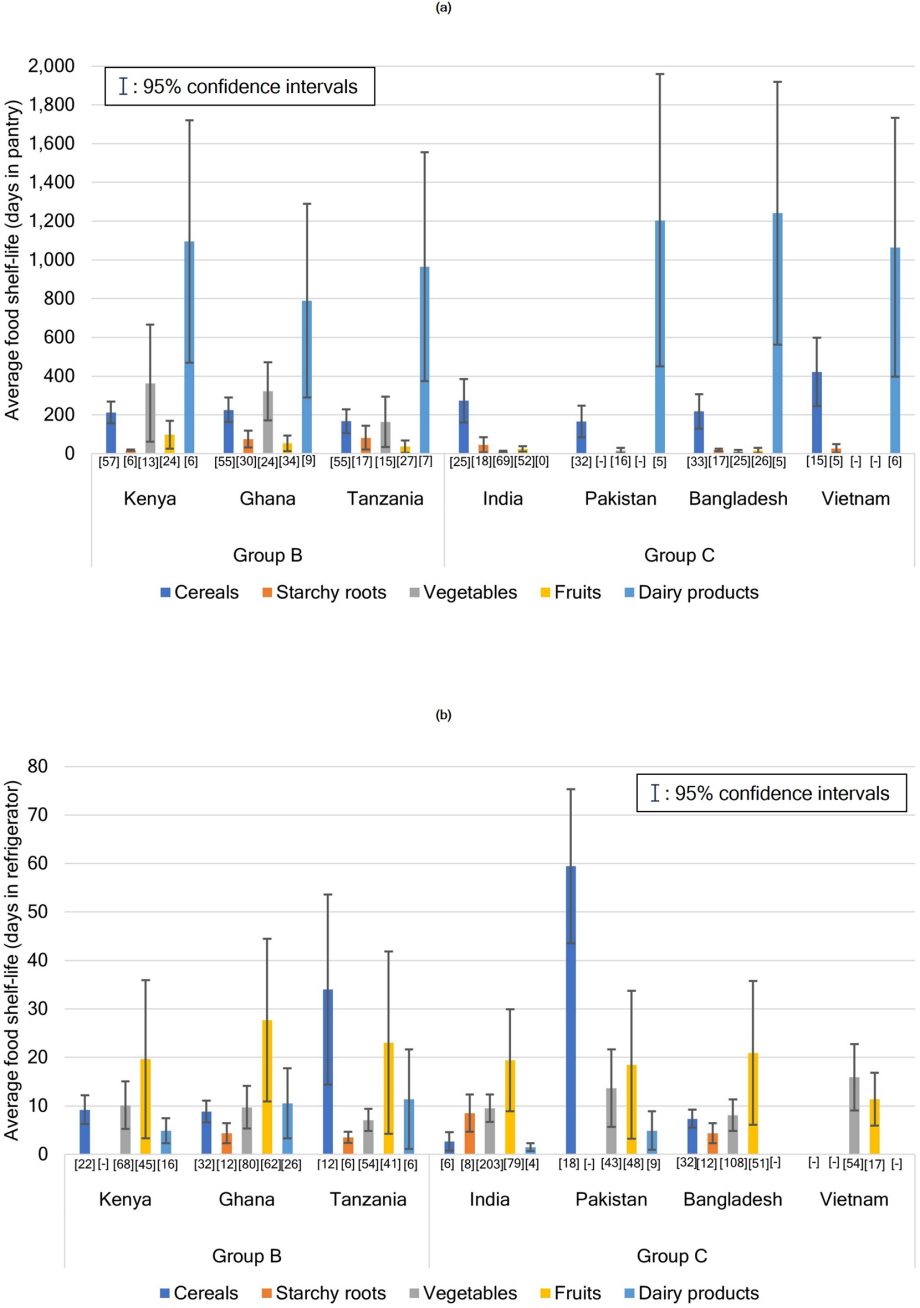
Food shelf life in pantry and refrigerator storage in countries for which data were available. (**a**) and (**b**) show the average shelf lives of food item groups in pantry and refrigerator storage, respectively, with error bars for 95% confidence intervals (95% CIs). Sample sizes are shown as numbers in parentheses under the horizontal axis. If the range of 95% CIs (error bars) reached negative values, the mean values were excluded from discussion and their sample sizes are shown as “-.” The complete data for the figure are provided in Supplementary Data S3.

**Fig. 5:**
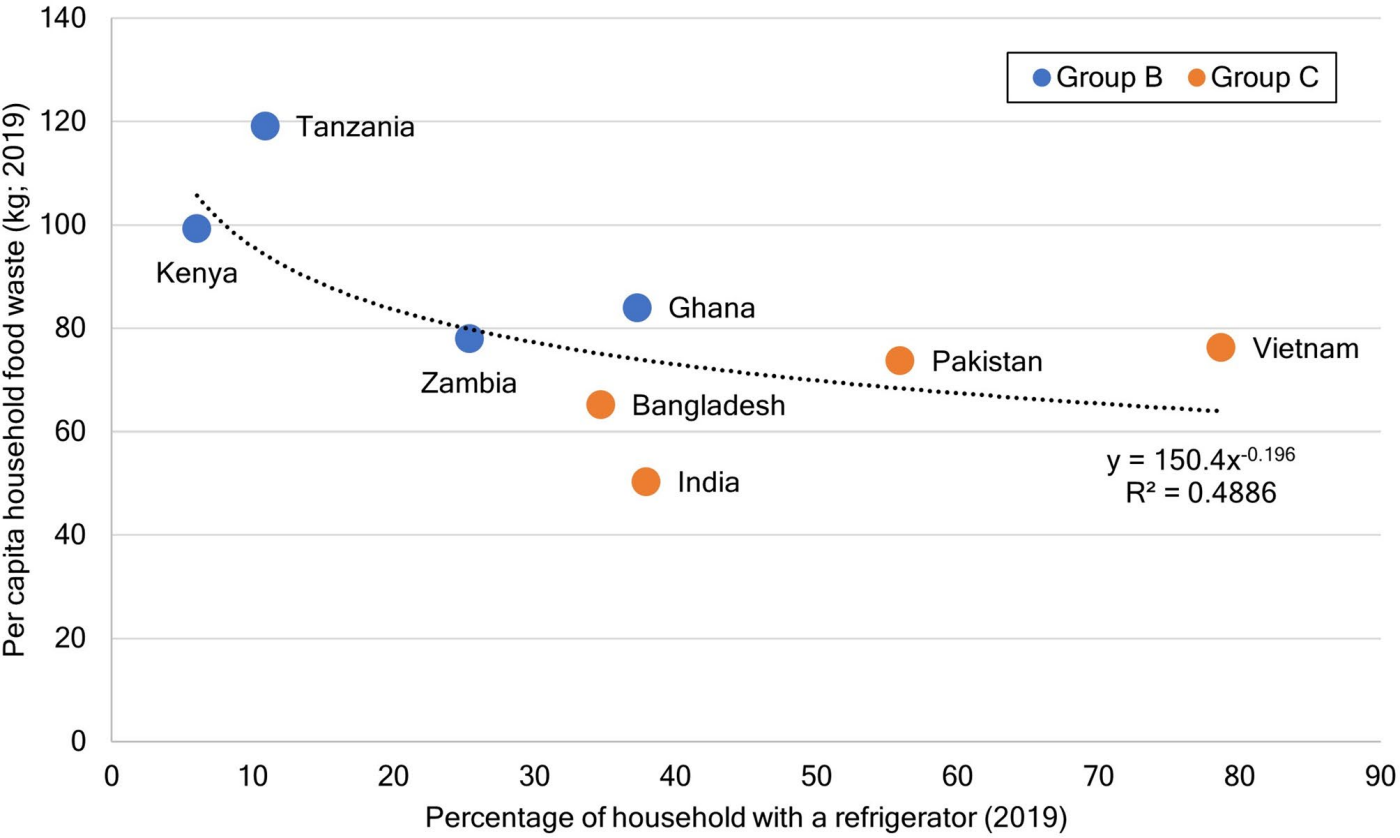
Correlation between household food waste and possession of refrigerators for thew target countries. A scatter diagram between the percentage of households with a refrigerator and per capita household food waste in 2019 for groups B and C, shown as blue and orange dots, respectively, was plotted. The percentage of households with refrigerators was compiled from data surveyed in 2019 in a database of Global Data Lab (https://globaldatalab.org/areadata/table/fridge/; accessed: July 11, 2023). For countries without survey data in 2019, the percentage of households with refrigerators was estimated with linear interpolation or extrapolation using results of the closest two survey years from 2019.

HFW accrues in daily life routines such as shopping, storing, cooking, and eating^14,18,25^. Storage is considered the most critical practice for food waste reduction because foods with short shelf lives are more likely to be wasted^14^, and food perishability depends on correct food storage^6,13,14^. Therefore, the difference in food shelf life may cause food waste generation in households in groups B and C; in other words, the shelf life of the primary food in group C exceeds that in group B. With reference to the food keeper database developed by the United States of America (https://www.foodsafety.gov/keep-food-safe/foodkeeper-app) and literature reviews^47–50^, in this study, we developed a food shelf-life dataset according to storage (pantry, refrigerator, and freezer) based on the following available items: cereals, starchy roots, vegetables, fruits, and dairy products in the FCTs of seven countries (Supplementary Data S3). Food shelf life using freezer storage did not show a considerable difference between groups B and C, but that using pantry and refrigerator storage showed differences. In pantry storage (Fig. 4a), dairy products such as powdered milk or processed products (such as cheese) showed the longest shelf life (790–1,241 days on average). Starchy roots had a shorter shelf life than vegetables, which are typically dried or canned, in group B, but had a longer shelf life than vegetables and fruits in group C. In contrast, in refrigerator storage (Fig. 4b), the shelf life of vegetables and fruits was longer than that of starchy roots. Based on these results, starchy roots for group B and vegetables and fruits for group C may be the primary constituents of HFW in case of pantry storage; the particularly short shelf life of starchy roots may increase the per capita HFW of group B. Furthermore, refrigeration probably increases the shelf life of vegetables and fruits compared with that of starchy roots in group C, whereas the average shelf life of starchy roots remains shorter than that of vegetables and fruits even after refrigeration. In addition, the correlation between the percentage of households with refrigerators and per capita HFW of the eight countries in groups B and C was not significant (*p* = 0.17). The results show a trend, likely fitting a power approximation expression with R^2^ = 0.49 (Supplementary Data S1), where per capita household food wastage might be reduced because of refrigeration (Fig. 5).

## Discussion

By focusing on FEI instead of food waste, we comprehensively clarify EGA results, especially negative values, which are unreasonable in terms of food waste. Although previous studies have proposed methods to treat or correct such negative EGA values^37,38^, we show that EGA results can be appropriately defined as negative FEI. Our study thus advances the search for an indicator for SDG 2.1. In addition, we could categorize countries based on the temporal variation in FEI (Table 1) by setting a base year (2005) that follows the reference model^36^. However, a limitation of such an approach is that the results for years prior to 2005 could not be utilized due to the lack of validation.

**Table 1:** Categories of target countries.

No.	Country code^#^	Confidence in household food waste estimates^3^	Temporal variation of FEI	World Bank income group^*^
1	AUS	High	HE	HIC
2	AUT	High	HE	HIC
3	BEL	Medium	HE	HIC
4	BGD	Medium	DE	LMC
5	BLZ	Medium	LE	LMC
6	BRA	Medium	HE	UMC
7	CAN	High	HE	HIC
8	CHN	Medium	HE	UMC
9	COL	Medium	HE	UMC
10	DEU	High	HE	HIC
11	DNK	High	HE	HIC
12	ESP	Medium	HE	HIC
13	EST	Medium	HE	HIC
14	ETH	Medium	LD	LIC
15	FIN	Medium	HE	HIC
16	FRA	Medium	HE	HIC
17	GBR	High	HE	HIC
18	GEO	Medium	HE	UMC
19	GHA	High	LD	LMC
20	GRC	Medium	LE	HIC
21	HUN	Medium	HE	HIC
22	IDN	Medium	HE	LMC
23	IND	Medium	DE	LMC
24	IRL	Medium	HE	HIC
25	IRQ	Medium	HE	UMC
26	ISR	Medium	HE	HIC
27	ITA	Medium	LE	HIC
28	JPN	Medium	HE	HIC
29	KEN	Medium	LD	LMC
30	LBN	Medium	HE	UMC
31	LKA	Medium	HE	LMC
32	LUX	Medium	HE	HIC
33	MEX	Medium	HE	UMC
34	MLT	High	HE	HIC
35	MYS	Medium	HE	UMC
36	NGA	Medium	HE	LMC
37	NLD	High	HE	HIC
38	NOR	High	HE	HIC
39	NZL	High	HE	HIC
40	PAK	Medium	DE	LMC
41	POL	Medium	HE	HIC
42	RUS	Medium	HE	UMC
43	RWA	Medium	LD	LIC
44	SAU	High	HE	HIC
45	SVN	Medium	HE	HIC
46	SWE	High	HE	HIC
47	TZA	Medium	LD	LMC
48	USA	High	HE	HIC
49	VNM	Medium	DE	LMC
50	ZAF	Medium	HE	UMC
51	ZMB	Medium	LD	LMC

^#^ ISO3166-1; ^*^ https://datahelpdesk.worldbank.org/knowledgebase/articles/906519-world-bank-country-and-lending-groups.

There have been two major proposals regarding food waste: (i) food waste is proportional to the national development level^11,19,20,37,51,52^ or (ii) there is a negligible correlation between food waste and national development level^21,22^. In the present study, the national income level, in which FEI is logarithmically proportional to Eq. (1), was not confirmed to be correlated with HFW (Figs. 1 and 2), consistent with the findings of previous studies^21,22^. However, in lower-middle-income countries, the per capita HFW of group B with a negative FEI was remarkably higher than that of group C, whose FEI changed from negative to positive (Fig. 2). This pattern was observed although the per capita GDP in 2019, based on the mean value as shown in Supplementary Data S4, of group B (2,570 real International$ in 2005) was approximately half of that of group C (5,035 real International$ in 2005). This result indicates that HFW may decrease with an increase in the national income level in some lower-middle income counties, although previous studies reported that such growth in the per capita income should increase food waste^7,11,19,20,37,52^. We assume that this result could be due to the fact that the food shelf life in group C is longer than that in group B owing to a higher percentage of households with a refrigerator (Figs. 4 and 5) and a growing income level. Additionally, a growth in income possibly reduces HFW by increasing the utilization of food service sectors (food-away-from-home)^7,13,21,18,52,53^ in group C compared with that in group B. However, as food waste from food services in Bangladesh in group C (3 kg/capita/year) was lower than that in Kenya in group B (31 kg/capita/year), whose food waste estimates in the food service sectors are relatively reliable^21^, food-away-from-home does not necessarily contribute to HFW reduction in group C.

Our study implies that lower-middle-income countries, categorized as group B, could achieve a reduction in HFW (SDG 12.3) as well as improve food security (SDG 2.1). Previous studies have proposed theoretical synergies between SDG 12.3 and other goals such as SDG2 (zero hunger)^3,5^, SDG3 (good health and well-being)^35^, SDG4 (quality education)^10^, SDG6 (clean water and sanitation)^5^, SDG13 (climate action)^5^, SDG15 (life on land)^5,35^, SDG16 (peace, justice and strong institutions)^10^, and SDG17 (partnerships for the goals)^10^. An increase in the number of households with refrigerators may substantially promote these achievements. However, these implications could not be verified because the related scientific knowledge and data on HFW for lower-middle-income countries are limited, as are data on food losses^12^. Data availability on HFW is particularly constrained, and reliable data (high or medium confidence estimates) are only available for 12 lower-middle-income countries (Fig. 2). Of these, the only country with high confidence estimates is Ghana, indicating that the data for this country are suitable for tracking national levels of HFW^21^. For countries without data, a previous study extrapolated average income and regional group data from similar countries^21^.

We consider two limitations in the calculation of FEI, which are related to subsistence farming and demographic composition. A lack of food from subsistence farming^36,54–56^ may cause a negative FEI by underestimating food energy supply for lower-middle-income countries. In addition, it is uncertain whether subsistence farmers consume their own non-marketable products without generating waste compared with other marketable food because self-harvested food may both reduce and increase food waste^18^. FEI is based on a population aged 15 years and older, excluding pregnant and lactating women^36^. In countries with a relatively young demographic composition, a positive FEI may thus be underestimated, whereas a negative FEI may be overestimated because actual food energy requirements may be lower than those estimated^57,58^. Based on World Bank Open Data, the average percentages of the population under 15 years of age in group B were 44% (2005) and 41% (2019), whereas those in group C were 34% (2005) and 29% (2019); therefore, the negative FEI of group B might be overestimated. However, as such countries are presumed to have a high percentage of pregnant and lactating women, who need additional food energy^57,58^, underestimation of the results is also possible. Therefore, particularly for the 19 lower-middle-income countries (Table 2), with food supply below the required limit despite improvement compared with the base year, and for some areas lacking high-quality data^11,17,22^, it is important to collect accurate data on food supply from subsistence farming and consumer behavior toward HFW from subsistence products. This could be achieved via collaboration with international initiatives that support regular surveys of farm households for food loss in lower-income and lower-middle-income countries^12^. It is likewise important to collect data on food storage methods in households, food energy requirements of children aged ≤ 15 years and pregnant and lactating women, and food waste quantity to verify the synergy between SDG 12.3 and SDG 2.1.

**Table 2:** Lower-middle-income countries recommended for future investigation to verify the synergy between sustainable development goals 12.3 and 2.1.

No	Country code	Name of country	FEI (kcal/day/cap; 2005)	FEI (kcal/day/cap; 2019)
1	BEN	Benin	− 289	− 154
2	CMR	Cameroon	− 179	− 82
3	COM	Comoros	− 302	− 256
4	GHA	Ghana	− 503	− 192
5	HTI	Haiti	− 204	− 166
6	KEN	Kenya	− 417	− 255
7	KIR	Kiribati	− 379	− 332
8	LSO	Lesotho	− 413	− 261
9	NPL	Nepal	− 405	− 99
10	PNG	Papua New Guinea	− 468	− 245
11	SEN	Senegal	− 207	− 96
12	SLB	Solomon Islands	− 386	− 260
13	STP	São Tomé and Principe	− 238	− 55
14	TJK	Tajikistan	− 392	− 46
15	TLS	Timor-Leste	− 515	− 255
16	TZA	Tanzania	− 434	− 192
17	VUT	Vanuatu	− 225	− 169
18	ZMB	Zambia	− 254	− 40
19	ZWE	Zimbabwe	− 642	− 521

## Methods

### Definition of FEI and its calculation

In this study, we defined FEI as energy excess or deficit of food relative to the energy requirements in humans. Human energy requirement is defined as the amount of food energy needed to balance energy expenditure to maintain body size, body composition, and a level of necessary and desirable physical activity consistent with long-term good health^57^. Humans should live a long and healthy life if food energy intake balances with these requirements (FEI = 0). Therefore, FEI can be defined as an indicator for food energy intake or supply that is not recommended for a long-term healthy life. Thus, FEE, which potentially leads to overweight and obesity, is accounted for by a positive FEI if food energy supply is higher than the energy requirement, and food waste is considered an indicator of oversupply^20,36–38,58^. In contrast, FED, which typically causes undernourishment and starvation, is quantified as negative FEI if food energy supply is not consistent with the food requirements^58^.

The national FEI was estimated using a log-linear model based on the EGA^36,59^. Although a previous study^36^ presented two models, one based on the annual per capita actual individual consumption and one based on the GDP, we adopted the second model, shown as Eq. (1), because of interannual data accessibility.

1 $$fs = {\text{ }}557{\text{ }} \times {\text{ }}\ln \left( {gdp} \right){\text{ }}{-}{\text{ }}4,537$$

where *fs* and *gdp* are the per capita FEI (kcal/day, average) and per capita GDP (real International $ in 2005), respectively. Presumably, a nation has an on average sufficient food supply if *fs* is positive, while a nation faces an FED if *fs* is negative. As the original model defines the base year as 2005, the per capita GDP was calculated using the GDP (current LCU) for 2005, the GDP (constant LCU) for 2005 and the target year, the PPP conversion factor for the GDP (LCU per international $) for 2005, and the total population for the target year, by nation, based on World Bank Open Data (https://data.worldbank.org/; accessed: April 12, 2022). Finally, 3,034 values of per capita FEI (kcal/day) for 185 − 191 countries worldwide from 2005 to 2020 were calculated (Supplementary Data S4). In addition, the previous paper based our analysis on presented lower- and upper-case models based on 95% confidence intervals^36^; however, in the present study, the results of those models were excluded, as they were found to deviate from reality. That is, the lower-case model indicated an FED for developed countries such as Australia, Austria, Belgium, Denmark, Finland, and Japan between 2005 and 2020, whereas the upper-case model indicated no country with an FED from 2008 to 2016.

### Collecting nation-specific data of per capita HFW

Nation-specific data on per capita HFW were collected from an international dataset for 2019^21^ . The data were estimated based on a review of numerous data points from direct measurements of food waste generation in countries for which data were available. This enabled us to finally cover food waste generation (kg/capita/year) in 215 countries, including bias correction and extrapolation for countries without data. As the accuracy or representativeness of data differs by country, we referred to the following categories of countries in the dataset: high, medium, low, and very low confidence; we focused on countries in the high- and medium-confidence groups (52 countries). This is because food waste estimates, based on country-specific data, for the high- and medium-confidence groups were more accurate and representative than those for the low- and very low-confidence groups referring to data in similar or neighboring countries. In addition, the dataset included data on food waste at the household level, as well as at the food service and retail levels; however, we utilized only data on HFW in this study because the number of countries with high or medium confidence at the food service and retail levels (23 countries for both) was small compared with the confidence of 52 countries at the household level. The related data points may be representative of only 32 and 14% of the global population, respectively, whereas those of HFW may represent 75% of the global population^21^.

## Selecting and categorizing comparable countries

We defined 2019 as the target year based on the availability of data for FEI and HFW. Correspondingly, 2005 was defined as the base year by following the log-linear model^36^ to evaluate temporal variations in FEI. Thereafter, 51 countries, which had per capita HFW for the target year (in 52 countries) and per capita FEI estimates for the base and target year (in 191 and 187 countries, respectively), were selected as comparable countries.

Based on the temporal variation in FEI between the base and target years, the target countries were categorized into five groups: higher food energy deficit (HD), lower food energy deficit (LD), deficit to excess of food energy (DE), higher food energy excess (HE), and lower food energy excess (LE). HD and LD countries have insufficient food energy supply in both base and target years. The HD group has a more serious shortage in the target than in the base year, whereas the LD group has a less serious shortage in the target than in the base year. Similarly, HE and LE countries have sufficient food supply, while the HE group has a more excessive food supply in the target than in the base year. The LE group has a lower food supply in the target than in the base year. Finally, DE countries have sufficient food supply in the target year although the supply was insufficient in the base year.

We further categorized the target countries into high-income countries (HICs), upper-middle-income countries (UMCs), lower-middle-income countries (LMCs), and low-income countries (LICs) based on the World Bank income groupings for 2019 (https://datahelpdesk.worldbank.org/knowledgebase/articles/906519-world-bank-country-and-lending-groups). The attribution of target countries is summarized in Table 1.

### Statistical analysis

We used Microsoft^®^ Excel^®^ for Microsoft 365 MSO (version 2401 build 16.0.17231.20236) 64 bits for single linear-regression analysis (Figs. 1 and 5) and for performing the *t-*test to determine significant differences in HFW between groups B and C (Fig. 2). The input data and outputs for the single linear-regression analyses are provided as Supplementary Data S1. The p-value for the difference between groups B and C was computed using the T.TEST function as follows: =T.TEST (array-1, array-2, 2,3) with array-1=[84.0075, 77.92, 99.2385369915733, 119.08611] and array-2=[50.3214780952381, 65.124075, 73.638965625, 76.1616925875]. The p-value between groups C and D was computed using the T.TEST function as follows: =T.TEST (array-2, array-3, 2,3) with array-3=[75.86571225, 77.36981175, 188.797725].

## Electronic supplementary material

Below is the link to the electronic supplementary material.

Supplementary Material 1

Supplementary Material 2

Supplementary Material 3

Supplementary Material 4

Supplementary Material 5

Supplementary Material 6

## Data Availability

Data are provided within the manuscript or supplementary information files.

## References

[1] Tian, P. et al. Keeping the global consumption within the planetary boundaries. *Nature* **635**, 625–630. 10.1038/s41586-024-08154-w (2024). 39537917 10.1038/s41586-024-08154-w PMC11578892

[2] United Nations. 64 (United Nations, NY, 2022).The Sustainable Development Goals (2022).

[3] Chang, J. et al. Reconciling regional nitrogen boundaries with global food security. *Nat. Food*. **2**, 700–711. 10.1038/s43016-021-00366-x (2021). 37117470 10.1038/s43016-021-00366-x

[4] Springmann, M. et al. Options for keeping the food system within environmental limits. *Nature* **562**, 519–525. 10.1038/s41586-018-0594-0 (2018). 30305731 10.1038/s41586-018-0594-0

[5] Hasegawa, T., Havlík, P., Frank, S., Palazzo, A. & Valin, H. Tackling food consumption inequality to fight hunger without pressuring the environment. *Nat. Sustain.* **2**, 826–833. 10.1038/s41893-019-0371-6 (2019).

[6] Beretta, C., Stoessel, F., Baier, U. & Hellweg, S. Quantifying food losses and the potential for reduction in Switzerland. *Waste Manage.* **33**, 764–773 (2013). 10.1016/j.wasman.2012.11.007 23270687

[7] Xue, L. et al. Missing food, missing data? A critical review of global food losses and food waste data. *Environ. Sci. Technol.* **51**, 6618–6633 (2017). 28492315 10.1021/acs.est.7b00401

[8] Springmann, M., Clark, M. A., Rayner, M., Scarborough, P. & Webb, P. The global and regional costs of healthy and sustainable dietary patterns: a modelling study. *Lancet Planet. Health*. **5**, e797–e807. 10.1016/S2542-5196(21)00251-5 (2021). 34715058 10.1016/S2542-5196(21)00251-5 PMC8581186

[9] Fesenfeld, L., Rudolph, L. & Bernauer, T. Policy framing, design and feedback can increase public support for costly food waste regulation. *Nat. Food*. **3**, 227–235. 10.1038/s43016-022-00460-8 (2022). 37117636 10.1038/s43016-022-00460-8

[10] Some, S., Roy, J., Chatterjee, J. S. & Butt, M. H. Low demand mitigation options for achieving sustainable development goals: role of reduced food waste and sustainable dietary choice. *J. Clean. Prod.* **369**, 133432. 10.1016/j.jclepro.2022.133432 (2022).

[11] Gatto, A. & Chepeliev, M. Global food loss and waste estimates show increasing nutritional and environmental pressures. *Nat. Food*. **5**, 136–147. 10.1038/s43016-023-00915-6 (2024). 38287151 10.1038/s43016-023-00915-6

[12] Fabi, C., Cachia, F., Conforti, P. & English, A. Rosero Moncayo, J. Improving data on food losses and waste: from theory to practice. *Food Policy*. **98**, 101934. 10.1016/j.foodpol.2020.101934 (2021).

[13] Hoehn, D. et al. A critical review on food loss and waste quantification approaches: is there a need to develop alternatives beyond the currently widespread pathways? *Resour. Conserv. Recy*. **188**, 106671. 10.1016/j.resconrec.2022.106671 (2023).

[14] Hebrok, M. & Boks, C. Household food waste: drivers and potential intervention points for design – An extensive review. *J. Clean. Prod.* **151**, 380–392. 10.1016/j.jclepro.2017.03.069 (2017).

[15] Thyberg, K. L., Tonjes, D. J. & Gurevitch, J. Quantification of food waste disposal in the united States: A meta-analysis. *Environ. Sci. Technol.* **49**, 13946–13953. 10.1021/acs.est.5b03880 (2015). 26551283 10.1021/acs.est.5b03880

[16] Teigiserova, D. A., Hamelin, L. & Thomsen, M. Towards transparent valorization of food surplus, waste and loss: clarifying definitions, food waste hierarchy, and role in the circular economy. *Sci. Total Environ.* **706**, 136033. 10.1016/j.scitotenv.2019.136033 (2020). 31855638 10.1016/j.scitotenv.2019.136033

[17] Ardra, S. & Barua, M. K. Halving food waste generation by 2030: the challenges and strategies of monitoring UN sustainable development goal target 12.3. *J. Clean. Prod.* **380**, 135042. 10.1016/j.jclepro.2022.135042 (2022).

[18] Schanes, K., Dobernig, K. & Gözet, B. Food waste matters - A systematic review of household food waste practices and their policy implications. *J. Clean. Prod.* **182**, 978–991. 10.1016/j.jclepro.2018.02.030 (2018).

[19] FAO. Global food losses and food waste – Extent, causes and prevention. Rome, (2011).

[20] Tilman, D. & Clark, M. Global diets link environmental sustainability and human health. *Nature* **515**, 518–522. 10.1038/nature13959 (2014). 25383533 10.1038/nature13959

[21] United Nations Environment Programme. Food Waste Index Report 2021. Nairobi, (2021).

[22] Dou, Z. & Toth, J. D. Global primary data on consumer food waste: rate and characteristics – A review. *Resour. Conserv. Recy*. **168**, 105332. 10.1016/j.resconrec.2020.105332 (2021).

[23] Conrad, Z. et al. Relationship between food waste, diet quality, and environmental sustainability. *PLoS One*. **13**, e0195405. 10.1371/journal.pone.0195405 (2018). 29668732 10.1371/journal.pone.0195405 PMC5905889

[24] Conrad, Z. & Blackstone, N. T. Identifying the links between consumer food waste, nutrition, and environmental sustainability: a narrative review. *Nutr. Rev.* **79**, 301–314. 10.1093/nutrit/nuaa035 (2020). 10.1093/nutrit/nuaa035 32585005

[25] Block, L. G. et al. The squander sequence: Understanding food waste at each stage of the consumer decision-making process. *J. Public. Policy Mark.* **35**, 292–304. 10.1509/jppm.15.132 (2016).

[26] Spada, A., Conte, A. & Del Nobile, M. A. The influence of shelf life on food waste: A model-based approach by empirical market evidence. *J. Clean. Prod.* **172**, 3410–3414. 10.1016/j.jclepro.2017.11.071 (2018).

[27] Lundqvist, J., Fraiture, C. D. & Molden, D. *Saving Water: from Field To Fork. Curbing Losses and Wastage in the Food Chain* (SIWI, 2008).

[28] Ulf, S., Frida, A., Davis, J. & Per-Olow, S. Home transport and wastage: environmentally relevant household activities in the life cycle of food. *Ambio* **34**, 371–375 (2005). 16092271 10.1639/0044-7447(2005)034[0371:htawer]2.0.co;2

[29] Romani, S., Grappi, S., Bagozzi, R. P. & Barone, A. M. Domestic food practices: A study of food management behaviors and the role of food Preparation planning in reducing waste. *Appetite* **121**, 215–227. 10.1016/j.appet.2017.11.093 (2018). 29155173 10.1016/j.appet.2017.11.093

[30] Prokopov, T. & Tanchev, S. *Methods of Food Preservation. Food Safety: A Practical and Case Study Approach 3–25* (Springer US, 2007).

[31] Reyes, V. & Cahill, E. Mis Solval, K. E. The potential for reducing food waste through shelf-life extension: actionable insights from data digitization. *Sustainability* **16**, 2986 (2024).

[32] Cavaliere, A. & Ventura, V. Mismatch between food sustainability and consumer acceptance toward innovation technologies among millennial students: the case of shelf life extension. *J. Clean. Prod.* **175**, 641–650. 10.1016/j.jclepro.2017.12.087 (2018).

[33] Hammond, S. T. et al. Food spoilage, storage, and transport: implications for a sustainable future. *BioScience* **65**, 758–768. 10.1093/biosci/biv081 (2015).

[34] Tian, P. et al. Higher total energy costs strain the elderly, especially low-income, across 31 developed countries. *Proc. Natl. Acad. Sci.* 121, e2306771121 (2024). 10.1073/pnas.2306771121 10.1073/pnas.2306771121 PMC10962987 38466846

[35] Guo, Y. et al. Global food loss and waste embodies unrecognized harms to air quality and biodiversity hotspots. *Nat. Food*. **4**, 686–698. 10.1038/s43016-023-00810-0 (2023). 37550539 10.1038/s43016-023-00810-0

[36] Verma, M. B., de Vreede, L., Achterbosch, T. & Rutten, M. M. Consumers discard a lot more food than widely believed: estimates of global food waste using an energy gap approach and affluence elasticity of food waste. *PLoS One*. **15**, e0228369. 10.1371/journal.pone.0228369 (2020). 32049964 10.1371/journal.pone.0228369 PMC7015318

[37] Lopez Barrera, E. & Hertel, T. Global food waste across the income spectrum: implications for food prices, production and resource use. *Food Policy*. **98**, 101874. 10.1016/j.foodpol.2020.101874 (2021).

[38] Bodirsky, B. L. et al. The ongoing nutrition transition thwarts long-term targets for food security, public health and environmental protection. *Sci. Rep.* **10**, 19778. 10.1038/s41598-020-75213-3 (2020). 33208751 10.1038/s41598-020-75213-3 PMC7676250

[39] Godfray, H. C. et al. Food security: the challenge of feeding 9 billion people. *Science* **327**, 812–818. 10.1126/science.1185383 (2010). 20110467 10.1126/science.1185383

[40] Welch, B. L. The significance of the difference between two means when the population variances are unequal. *Biometrika* **29**, 350–362. 10.1093/biomet/29.3-4.350 (1938).

[41] Welch, B. L. The generalization of ‘STUDENT’S’ problem when several different population varlances are involved. *Biometrika* **34**, 28–35. 10.1093/biomet/34.1-2.28 (1947). 20287819 10.1093/biomet/34.1-2.28

[42] Bentham, J. et al. Multidimensional characterization of global food supply from 1961 to 2013. *Nat. Food*. **1**, 70–75. 10.1038/s43016-019-0012-2 (2020). 32002520 10.1038/s43016-019-0012-2 PMC6992427

[43] FAO & Government of Kenya. Kenya Food Composition Table 254. Nairobi, (2018).

[44] Shaheen, N. et al. *Food Composition Table for Bangladesh. Report No. 978984337522-3, Institute of Nutrition and Food Science, Centre for Advanced Research in Sciences* (University of Dhaka, Dhaka, 2013).

[45] Vincent, A. & FAO/INFOODS Food Composition Table for Western Africa. User Guide & Condensed Food Composition Table (FAO, Rome, 2020). (2019).

[46] Ministry of Health & Nutritional Institute. *Vietnamese Food Composition Table 527* (Medical Publishing House, 2007).

[47] International Fund for Agricultural Development (IFAD). *& FAO. The World Cassava economy - Facts, Trends and Outlook* (FAO and IFAD, 2000).

[48] Afriyie, E. et al. Determinants of household-level food storage practices and outcomes on food safety and security in Accra, Ghana. *Foods* 11, 3266 (2022). 10.3390/foods11203266 PMC9601939 37431014

[49] Ministry of Agriculture, L., Fisheries & Cooperative Republic of Kenya. Guidelines for sustainable healthy diets at household level, < (2021). https://kilimo.go.ke/wp-content/uploads/2021/01/Guidelines-for-sustaining-healthy-diets-at-household-level.pdf

[50] MILK-ED. Shelf-life of milk and milk products, milk-ed.eu/wp-content/uploads/2022/06/Shelf-life-and-storage-of-milk-and-milk-products_EN.pdf (2022).

[51] Gil, J. Going to waste. *Nat. Food*. **1**, 192–192. 10.1038/s43016-020-0067-0 (2020).

[52] Yu, Y. & Jaenicke, E. C. Estimating food waste as household production inefficiency. *Am. J. Agric. Econ.* **102**, 525–547. 10.1002/ajae.12036 (2020).

[53] Harvey, J., Nica-Avram, G., Smith, M., Hibbert, S. & Muthuri, J. Mapping the landscape of consumer food waste. *Appetite* **168**, 105702. 10.1016/j.appet.2021.105702 (2022). 34555494 10.1016/j.appet.2021.105702

[54] Svedberg, P. 841 Million Undernourished? *World Development* 27, 2081–2098 (1999). 10.1016/S0305-750X(99)00102-3

[55] Hawkesworth, S. et al. Feeding the world healthily: the challenge of measuring the effects of agriculture on health. *Philos. Trans. R Soc. B*. **365**, 3083–3097. 10.1098/rstb.2010.0122 (2010). 10.1098/rstb.2010.0122 PMC2935110 20713404

[56] FAO. *Food Balance Sheets A Handbook. 95* (FAO, 2001).

[57] FAO/WHO/UNU. *Human Energy Requirements. 96* (FAO, 2004).

[58] Hiç, C., Pradhan, P., Rybski, D. & Kropp, J. P. Food surplus and its climate burdens. *Environ. Sci. Technol.* **50**, 4269–4277. 10.1021/acs.est.5b05088 (2016). 27054575 10.1021/acs.est.5b05088

[59] Osner, R. Food wastage. *Nutr. Food Sci.* **82**, 13–16. 10.1108/eb058904 (1982).

