# Supplementary material for: Synergy between SDGs 12.3 and 2.1 in lower-middle-income countries through the lens of food waste and energy imbalance
Source: Sci Rep. 2025 May 23;15:17956. doi: 10.1038/s41598-025-01579-x (PMC12102298; doi:10.1038/s41598-025-01579-x)
Supplement: Supplementary file 3 — Supplementary Material 3 [file 41598_2025_1579_MOESM3_ESM.docx]

**Supplementary information for**

**Synergy between SDGs 12.3 and 2.1 in lower-middle-income countries through the lens of food waste and energy imbalance**

# **Supplementary Data S1 Output files of regression analysis for Figs. 1 and 5**

The input data and outputs for Figs. 1 and 5 are presented in the Excel files labeled “Supplementary Table S1a.xlsx” and “Supplementary Table S1b.xlsx,” respectively.

# **Supplementary Information S1 Food supply in target countries**

The mass-based food supply that can potentially be transformed to household food waste is summarized in Supplementary Figure S1. The figure shows per capita food supply for the eight countries in groups B and C, detailing data such as related to food supply quantity (kg/capita/year) per item group (https://www.fao.org/faostat/en/). The legend indicates FAOSTAT item group codes, as listed in Supplementary Table S1. Cereals, starchy roots, vegetables, fruits, and dairy products in the main text correspond to FAOSTAT item group codes 2905, 2907, 2918, 2919, and 2948, respectively.


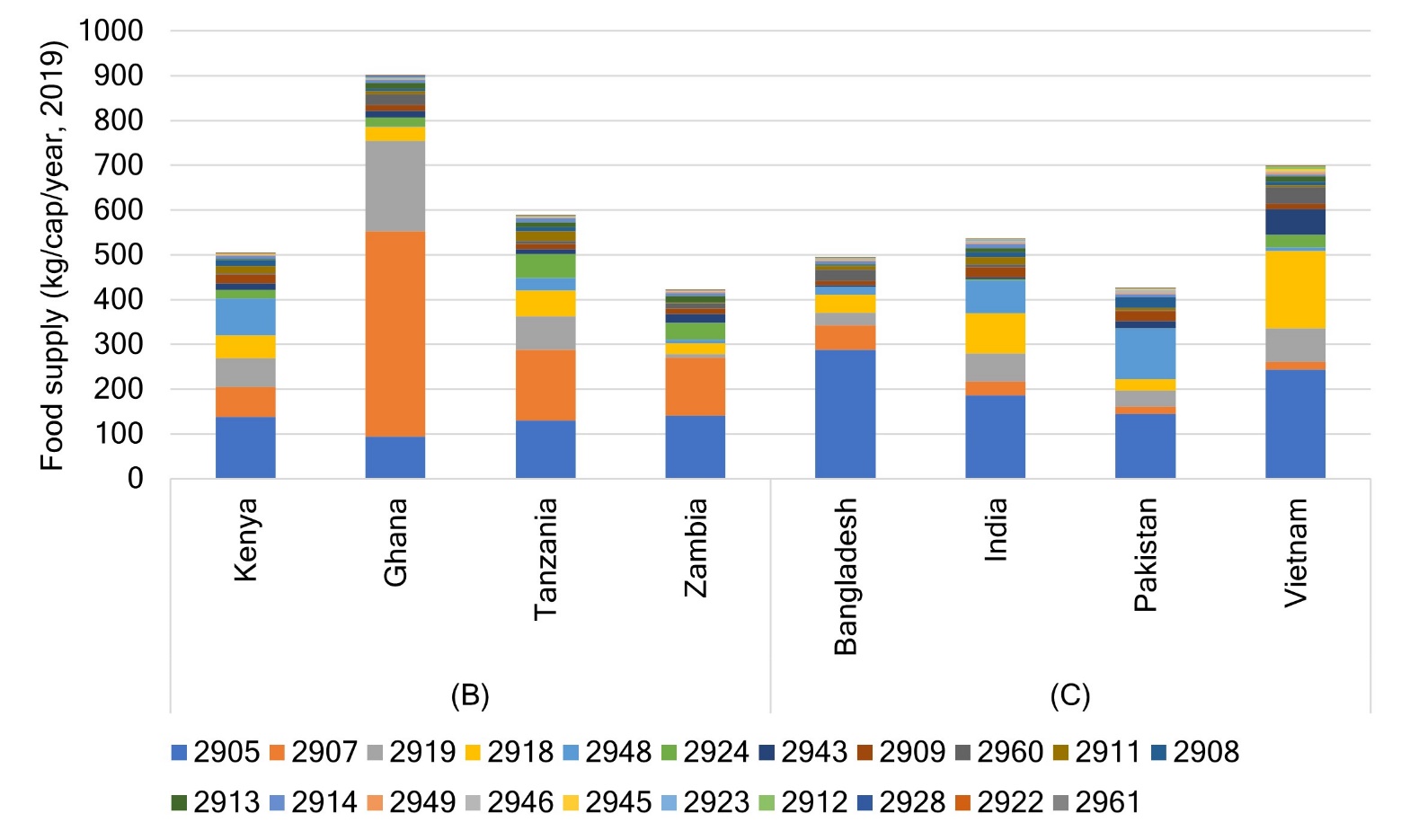


Supplementary Fig. S1 Per capita food supply in target countries (2019)

Supplementary Table S1 Item groups and their codes in the food balance sheet (FBS) in FAOSTAT

| Item group code | Item group | Item group code | Item group |
| --- | --- | --- | --- |
| 2905^*^ | Cereals - excluding beer | 2923 | Spices |
| 2907^*^ | Starchy roots | 2924 | Alcoholic beverages |
| 2908 | Sugar crops | 2928 | Miscellaneous |
| 2909 | Sugar and sweeteners | 2943 | Meat |
| 2911 | Pulses | 2945 | Offals |
| 2912 | Treenuts | 2946 | Animal fats |
| 2913 | Oilcrops | 2948^*^ | Milk - excluding butter |
| 2914 | Vegetable oils | 2949 | Eggs |
| 2918^*^ | Vegetables | 2960 | Fish and seafood |
| 2919^*^ | Fruits - excluding wine | 2961 | Aquatic products and others |
| 2922 | Stimulants |  |  |

Notes: Please refer to definitions and standards used in FAOSTAT (<https://www.fao.org/faostat/en/#definitions>). * Item groups discussed in the main text

# **Supplementary Data S2 Dataset for edible portion estimates of food in target countries for which data were available**

The actual dataset is separately compiled as “Supplementary Table S2.xlsx ” based on the food composition tables (FCTs) of Kenya^1^, Ghana (Western Africa^2^), Bangladesh^3^, and Vietnam^4^. The list consists of six columns: “Group” (B or C), “Country,” “Name of food item” (in the FCT), “Original item code” (of the FCT), “FBS_Item group code” (Supplementary Table S1), and “Edible portion.”

# **Supplementary Data S3 Dataset for food shelf-life in target countries for which data were available**

We compiled 2,776 data points on food shelf-life in Kenya, Ghana, Tanzania, India, Pakistan, Bangladesh, and Vietnam according to food item and storage. Among them, 2,718 data points were obtained from the FoodKeeper App (https://www.foodsafety.gov/keep-food-safe/foodkeeper-app), and the other 58 data points based on a literature review. As baked or dried cassava can be stored for several months^5^, the shelf-life of dried cassava in pantry storage was assumed to span 5 to 8 months. Cassava is mostly stored for 1 to 3 days in households in Ghana^6^, and based on this, we assumed that raw cassava can be stored for 1 to 3 days in a pantry. A guideline for Kenya (https://kilimo.go.ke/wp-content/uploads/2021/01/Guidelines-for-sustaining-healthy-diets-at-household-level.pdf) states that peeled cassava in water can be stored in a refrigerator for 1 month, which we thus used as the shelf-life of peeled cassava. The FoodKeeper App does not clarify, such as use-by date, the shelf-life of milk (plain or flavored) in a refrigerator; therefore, the shelf-life of unidentified items of raw milk was assumed to be 1 to 2 days based on the other report (https://milk-ed.eu/wp-content/uploads/2022/06/Shelf-life-and-storage-of-milk-and-milk-products_EN.pdf). The final dataset is provided in “Supplementary Table S3.xlsx” and consists of ten columns: “Group” (B or C), “Country,” “Name of food item” (in the FCT), “Original item code” (of the FCT), “FBS_Item group code” (Supplementary Table S1), “Storage” (pantry, refrigerator, or freezer), “Max-Min” (maximum or minimum value), “Food shelf-life (days),” “Reference item in FoodKeep App”, and “Other references.”

# **Supplementary Data S4 Estimated food energy imbalance and its input data of per capita GDP**

This dataset provides the results for the food energy imbalance (kcal/day/cap) estimated using log-linear models, in which we adopted the moderate-case formula because the results of the lower- and upper-case formulas^7^ were inconsistent with reality. Per capita GDP (real International$ in 2005) input data were calculated using World Bank Open Data (https://data.worldbank.org/) from 2005 to 2018 for 199 countries, as summarized in the file “Supplementary Table S4.xlsx.”

# **References**

1 FAO & Government of Kenya. Kenya Food Composition Tables. 254 (Nairobi, 2018).

2 Vincent, A. *et al.* FAO/INFOODS Food Composition Table for Western Africa (2019) User Guide & Condensed Food Composition Table. (FAO, Rome, 2020).

3 Shaheen, N. *et al.* Food Composition Table for Bangladesh. Report No. 978984337522-3, (Institute of Nutrition and Food Science, Centre for Advanced Research in Sciences, University of Dhaka, Dhaka, Bangladesh, 2013).

4 Ministry of Health & Nutritional Institute. Vietnamese Food Composition Table. 527 (Medical Publishing House, Hanoi, 2007).

5 International Fund for Agricultural Development (IFAD) & FAO. The world cassava economy - Facts, trends and outlook. (FAO and IFAD, Rome, 2000).

6 Afriyie, E. *et al.* Determinants of Household-Level Food Storage Practices and Outcomes on Food Safety and Security in Accra, Ghana. *Foods* **11**, 3266 (2022).

7 Verma, M. v. d. B., de Vreede, L., Achterbosch, T. & Rutten, M. M. Consumers discard a lot more food than widely believed: Estimates of global food waste using an energy gap approach and affluence elasticity of food waste. *PLOS ONE* **15**, e0228369 (2020). https://doi.org:10.1371/journal.pone.0228369
